# Dynamic SLAM Dense Point Cloud Map by Fusion of Semantic Information and Bayesian Moving Probability

**DOI:** 10.3390/s25175304

**Published:** 2025-08-26

**Authors:** Qing An, Shao Li, Yanglu Wan, Wei Xuan, Chao Chen, Bufan Zhao, Xijiang Chen

**Affiliations:** 1 Hubei Engineering Research Center for BDS-Cloud High-Precision Deformation Monitoring, Artificial Intelligence School, Wuchang University of Technology, Wuhan 430223, China; 120160450@wut.edu.cn; 2 School of Safety Science and Emergency Management, Wuhan University of Technology, Wuhan 430079, China; 330662@whut.edu.cn (S.L.); 308761@whut.edu.cn (B.Z.); chenxijiang@whu.edu.cn (X.C.); 3 School of Civil Engineering and Architecture, Wuhan University of Technology, Wuhan 430079, China; xuanwei1988@whut.edu.cn; 4 School of Resources and Environmental Engineering, Wuhan University of Technology, Wuhan 430079, China; chaoshu@cug.edu.cn

**Keywords:** dynamic scene, Bayesian moving probability, dense reconstruction

## Abstract

Most existing Simultaneous Localization and Mapping (SLAM) systems rely on the assumption of static environments to achieve reliable and efficient mapping. However, such methods often suffer from degraded localization accuracy and mapping consistency in dynamic settings, as they lack explicit mechanisms to distinguish between static and dynamic elements. To overcome this limitation, we present BMP-SLAM, a vision-based SLAM approach that integrates semantic segmentation and Bayesian motion estimation to robustly handle dynamic indoor scenes. To enable real-time dynamic object detection, we integrate YOLOv5, a semantic segmentation network that identifies and localizes dynamic regions within the environment, into a dedicated dynamic target detection thread. Simultaneously, the data association Bayesian mobile probability proposed in this paper effectively eliminates dynamic feature points and successfully reduces the impact of dynamic targets in the environment on the SLAM system. To enhance complex indoor robotic navigation, the proposed system integrates semantic keyframe information with dynamic object detection outputs to reconstruct high-fidelity 3D point cloud maps of indoor environments. The evaluation conducted on the TUM RGB-D dataset indicates that the performance of BMP-SLAM is superior to that of ORB-SLAM3, with the trajectory tracking accuracy improved by 96.35%. Comparative evaluations demonstrate that the proposed system achieves superior performance in dynamic environments, exhibiting both lower trajectory drift and enhanced positioning precision relative to state-of-the-art dynamic SLAM methods.

## 1. Introduction

Simultaneous localization and mapping (SLAM) technology has become one of the crucial technologies in this industry, with the ongoing development and popularity of mobile robots [1], specifically for obtaining the ability of robots to navigate and perform tasks in unknown settings autonomously. There is a great deal of interest in SLAM applications and research. Particularly, vision-based SLAM (VSLAM) technology stands out in that it can accurately pinpoint a robot’s location and create a comprehensive map of its environment at a minimal hardware cost, facilitating autonomous navigation and obstacle avoidance.

Visual SLAM (VSLAM) has garnered significant research attention in recent years due to its numerous advantages over laser-based SLAM systems, particularly its reliance on cost-effective camera sensors as the primary sensing modality, thereby driving the development of a series of mature VSLAM algorithms. For instance, some scholars have proposed and improved a series of algorithms, such as ORB-SLAM [2,3,4], RGB-D SLAM [5], LSD-SLAM [6], and Direct Sparse Mapping [7]. Among existing approaches, the ORB-SLAM series represents the most established feature point-based visual SLAM framework, widely regarded as a benchmark in the field. Campos et al. [4] proposed ORB-SLAM, the most advanced algorithm, which demonstrates robust real-time performance across diverse environments, including various indoor, outdoor, and multi-scale scenarios. Despite significant progress, VSLAM algorithms still face many challenges in practical applications, as the above algorithms are mainly designed for static environments to ensure the robustness and efficiency of the system. However, in real life, dynamic objects like people and animals are unavoidable, which makes it challenging for current algorithms to adjust to various situations. The fundamental cause of the aforementioned issue lies in the pervasive presence of moving objects within dynamic scenes. VSLAM algorithms typically extract both static and dynamic feature points, with the latter predominantly emerging from highly textured regions in the environment [8]. Consequently, the presence of dynamic feature points leads to a significant accumulation of erroneous feature correspondences, substantially degrading the stability and reliability of the SLAM system. In this case, faulty pose estimate and feature matching result in erroneous placement, variations in the creation of environmental maps, and the potential for many overlapping images inside the generated map. In order to enhance the precision and resilience of indoor positioning systems, researchers have employed weighting algorithms based on physical distances and clustering techniques for virtual access points [9]. This approach has also informed our own research. Thus, an increasing number of researchers in the field of mobile robot navigation focus on VSLAM algorithms that can adapt to dynamic scenes, minimize interference from dynamic objects, and improve positioning accuracy and system robustness [10].

To overcome the previously discussed challenges, an increasing number of researchers are considering dynamic objects in Visual SLAM (VSLAM) algorithms, thereby introducing the concept of dynamic VSLAM. Existing dynamic VSLAM employs a variety of strategies for handling dynamic objects, primarily including geometric-based and deep learning-based methods. Geometric-based methods identify dynamic elements by analyzing the motion consistency of objects in a scene. Optical flow and background removal are two popular methods for identifying and removing dynamic objects, which lessen their influence on the SLAM system. However, these approaches (including DM-SLAM [11] and ESD-SLAM [12]) remain limited in rapidly changing dynamic environments or complex scenes due to their fundamental dependence on the stability of visual features in image sequences. Conversely, convolutional neural networks (CNNs) are the main tool used by deep learning-based techniques to recognize dynamic objects in the scene. These techniques are effective in dynamic object recognition, but they frequently suffer from high computational costs and constrained real-time processing power. For example, DynaSLAM [13] combines ORB-SLAM2 with the Mask R-CNN instance segmentation algorithm to remove dynamic feature points. While this approach enhances SLAM accuracy in dynamic environments, its significantly increased computational complexity and resource demands impose practical constraints on real-time performance.

In summary, it offers BMP-SLAM, a semantic SLAM system based on object recognition for indoor dynamic settings, to effectively improve the positioning accuracy of VSLAM algorithms in dynamic situations and enable real-time operation. In order to minimize the influence of dynamic objects on the positioning and mapping of the SLAM system, BMP-SLAM combines object recognition with a motion probability propagation model based on the Bayesian theorem. Concurrently, it creates dense point cloud maps to help mobile robots accomplish tasks. The following is a summary of this paper’s primary contributions:

(1) To address the issues of poor positioning accuracy and robustness of SLAM systems in dynamic environments, BMP-SLAM integrates YOLOv5 to construct a dynamic object detection thread, which can identify and process moving objects in real-time and efficiently and significantly improves the positioning accuracy and robustness of the SLAM system in a dynamic environment.

(2) YOLOv5 may suffer from false positives and missed detection, leading to incomplete removal of dynamic features. Additionally, the geometric redundancy in dynamic bounding boxes can result in erroneous elimination of static features within the detected regions. To address these limitations, we propose the Bayesian Moving Probability model for motion by combining historical information and time-series data. The model enables the dynamic estimation of key points not to rely on a single observation, but to enhance the judgment through accumulated data. This approach significantly improves the SLAM system’s ability to perceive and understand the dynamic environment.

(3) To fulfill the complex robot navigation task, the dense point cloud mapping thread is constructed, and the static dense point cloud map is constructed by combining the semantic information captured by the dynamic target detection thread and the Bayesian Moving Probability model to cull the dynamic feature points.

## 2. Related Work

In recent years, the robustness problem of SLAM systems in dynamic environments has become the focus of many researchers. The key to solving this problem lies in effectively identifying objects in dynamic environments and eliminating the impact of their feature points. Currently, there are many algorithms available for this purpose, which can be mainly categorized into geometry-based and deep learning-based approaches. Current research in deep learning-based approaches predominantly concentrates on semantic segmentation and object detection techniques for dynamic SLAM systems.

### 2.1. Geometry-Based Dynamic SLAM

Geometric-based approaches to improve dynamic SLAM focus on recognizing and processing objects in dynamic environments through geometric constraints and visual motion estimation. The core of these methods lies in distinguishing objects from static backgrounds by analysing their motion patterns, which in turn excludes the interference of dynamic objects in the SLAM process. Wang et al. [14] cluster the depth map and set thresholds after using geometric methods and mathematical models to identify discrepancies in feature points. The clustered region will be recognized as a dynamic object if its non-normal value is greater than the threshold, which will subsequently lead to the recognition of the dynamic target in the relevant region. Dai et al. [15] used the correlation of points to differentiate between points in static and dynamic scenes, which in turn led to the achievement of improving the robustness of SLAM systems in dynamic environments. Palazzolo [16] implemented an efficient direct tracking framework employing a Truncated Signed Distance Function (TSDF) that leverages color-encoded TSDF data for sensor pose estimation, while incorporating geometric filtering to remove dynamic objects. Kim et al. [17] constructed an approach for estimating a background model from a complex depth scene, based on which an energy-based approach was also used to estimate sensor self-motion by density visual odometry. This method specifically takes into account the effect of moving targets, which improves the accuracy of the estimation. Klappstein [18] et al. proposed a dynamic object detection approach based on optical flow motion analysis, enabling the identification of moving elements in the scene. Lu et al. [11] developed a novel approach that exploits the inherent discriminative characteristics between moving and static elements in dynamic environments. Their method selectively extracts static features from dynamic scenes to effectively eliminate feature mismatches across both static and dynamic scenarios. The main advantages of these methods are their generalisability to the environment and computational efficiency, and their ability to adapt autonomously in unknown environments without relying on a priori knowledge or large-scale training data. However, when there are fast-moving objects in the environment or the scene changes are extremely complex, it may be difficult to accurately distinguish between dynamic and static elements by relying only on geometric information.

### 2.2. Deep Learning-Based Dynamic SLAM

With the rapid advancement of deep learning technologies in recent years, deep learning-based approaches have been increasingly integrated into dynamic SLAM systems to enhance their robustness and perception capabilities. Deep learning techniques enable SLAM systems to achieve enhanced object recognition and classification accuracy by leveraging large-scale training datasets to learn sophisticated feature representations of environmental elements. When integrated with prior environmental knowledge, this approach enables precise identification and removal of dynamic regions within the scene. Thus, deep learning can effectively enhance the robustness of feature extraction in unstructured backgrounds, significantly improving the accuracy of loop closure in feature matching for SLAM systems. Currently, deep learning-based dynamic SLAM is mainly divided into two categories: semantic segmentation and object detection. For example, the Dyna-SLAM [13] integrates Mask R-CNN [19] for semantic segmentation into the base of ORB-SLAM2 and employs a multi-view geometric method based on depth thresholds for dynamic judgment. Li et al. [20] tracked dynamic key points in a Bayesian probabilistic estimation framework by fusing semantic segmentation and geometric constraint results. Yu et al. suggested DS-SLAM [21] combines motion consistency checking techniques with real-time semantic segmentation to increase positioning accuracy and lessen the impact of dynamic objects in dynamic environments. Additionally, in order to increase the positioning accuracy, Asaad et al. [22] present Wi-Lo, an innovative hybrid positioning framework that synergistically combines Wi-Fi fingerprinting with LoRa-based RSSI localization, leveraging their complementary strengths to address the limitations of conventional approaches. The Blitz-SLAM [23] utilizes BlitzNet to obtain object bounding boxes and masks, quickly distinguishing between static backgrounds and dynamic areas in images. By integrating the advantages of semantic and geometric information from masks, RGB, and depth images, the system can operate robustly in dynamic environments while generating clean and accurate global point cloud maps. Although the above methods can accurately recognise dynamic objects through semantic segmentation models, these models are usually complex and difficult to strike a balance between segmentation accuracy, system load and the number of detected categories. They often fail to achieve real-time operational results due to high computational costs and slow operation speeds.

However, the YOLO [24] (You Only Look Once) and SSD [25] (Single Shot Multibox Detector) are two target detection networks that achieve a good balance between speed and accuracy, and so are two popular choices in the construction of semantic SLAM systems currently. YOLO-SLAM proposed by Wu et al. [26] tightly couples the target detection method with the geometric constraint method, which filters the dynamic features in the detection region, and then uses the depth difference with random sample consistency (RANSAC) to distinguish the dynamic features. Nam and Gon-Woo proposed Dynamic Vins [27], which combines object detection and depth information for dynamic feature recognition, achieving results comparable to semantic segmentation. And Peng et al. proposed YDD-SLAM [10] to effectively identify and process dynamic feature points in dynamic environments by combining depth information and motion features to optimise VSLAM performance. Yu et al. proposed YG-SLAM [28], which uses YOLOv5 for object detection. By combining the prior information from object detection, it employs optical flow to detect the motion of feature points, finally removing the feature points of dynamic objects based on the pixel movement speed. Dynamic-SLAM [29] proposed by Xiao et al. proposes a leakage compensation algorithm based on the speed invariance of adjacent frames is used to solve the problem of low recall of SSD detection, which greatly reduces the pose estimation error due to incorrect matching by using a selective tracking algorithm in the tracking thread to deal with the feature points of dynamic objects. Wei et al. introduced DO-SLAM [30], which proposes an outlier detection mechanism combined with object detection to further determine the true motion status of potential dynamic objects, thereby enhancing the stability and positioning accuracy of SLAM systems in dynamic environments.

From the above analysis, it can be seen that the current SLAM algorithms for dynamic environments mainly include geometric methods and deep learning methods, which have their own advantages and disadvantages, as shown in Table 1. Despite the fact that these techniques have made SLAM systems more capable of operating in real-time in dynamic situations, they suffer from limited fault tolerance, an excessive dependence on target detection networks, and subpar performance in the face of false positives and missing detection. The paper proposes the Bayesian Moving Probability Model as a solution to these problems, which successfully makes up for YOLOv5’s limitations. Through the integration of temporal data and historical information, the model makes it possible to dynamically estimate key point estimation, not only based on a single observation but also through the improved judgment of accumulated historical data. This greatly enhances the VSLAM system’s capacity to comprehend and adjust to the changing environment.

## 3. Materials and Methods

### 3.1. System Architecture

In real-world applications, SLAM algorithms continue to face numerous obstacles, particularly in dynamic situations. Moving objects can have a significant impact on the stability and accuracy of SLAM systems in dynamic environments. In order to solve this problem, the research in this paper focuses mainly on reducing the negative impact of dynamic environments on the performance of the algorithms. ORB-SLAM3 has been leading other VSLAM algorithms due to its advantages such as efficient feature extraction, powerful closed-loop detection capability and effective relocation strategy. Therefore, the algorithms in this paper are mainly improved based on ORB-SLAM3 so as to achieve excellent performance even in dynamic environments.

**Figure 1:**
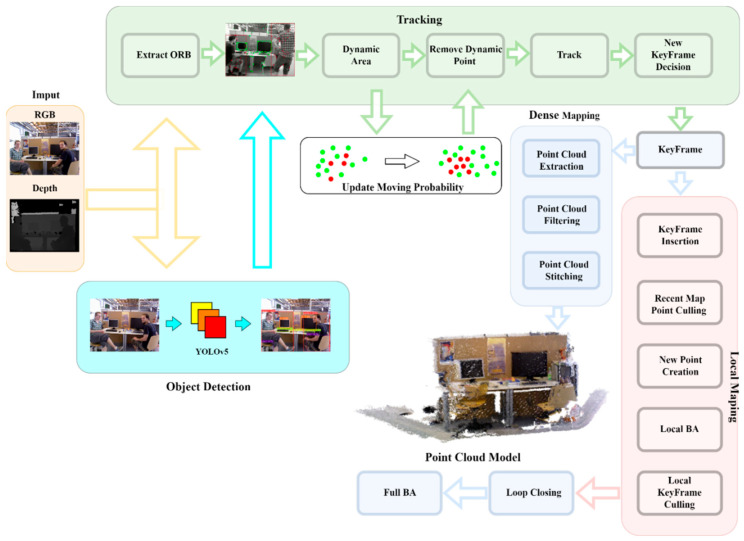
The framework diagram of BMP-SLAM.

ORB-SLAM3 primarily consists of four core threads: tracking, local mapping, loop closure detection, and map maintenance. Three new modules are added to the foundation of this study, as illustrated in Figure 1: dynamic object identification, the Bayesian Moving Probability model, and dense point cloud reconstruction. Firstly, the captured RGB image and depth image are input by the depth camera, and then, the tracking thread and the dynamic target detection thread are performed simultaneously. The tracking thread mainly performs ORB feature point extraction for the input RGB image to obtain the feature point information in the scene. The dynamic target detection thread mainly acquires semantic information in the RGB image through YOLOv5, such as the target’s label information and the coordinate information of the bounding box. Subsequently, based on the label information of the target, high dynamic objects, medium dynamic objects and low dynamic objects are identified, and the dynamic region is determined by combining the coordinate information of the ORB feature points with the coordinate information of the target bounding box. Then, the key points for different regions are propagated by the Bayesian motion probability propagation model to achieve the dynamic key point rejection, so that the ORB-SLAM3 algorithm does not retain them in the post-posture tracking and mapping process, as a way to improve the accuracy and robustness of the system in dynamic environments. Finally, the 3D information is extracted using the RGB images and depth images of the keyframes updated in the tracking thread, and a local point cloud with appropriate size and density is generated using voxel mesh filtering, transformed to the global coordinate system, and merged with the global map to create a dense point cloud map of the indoor 3D scene.

### 3.2. Dynamic Target Detection

To decrease the effect of dynamic targets on the SLAM system’s localization and map creation, semantic information is retrieved via object detection. This information is then fed into the BMP-SLAM system as semantic restrictions. To improve the system’s accuracy and robustness in dynamic situations. Since YOLOv5 employs CSPDarknet as its backbone network, it optimizes the transmission of feature maps via CSP (Cross Stage Partial connections), decreasing computation while retaining effective feature extraction. It also offers outstanding real-time performance, high detection accuracy, and can handle complicated interior dynamic settings; hence, BMP-SLAM employs YOLOv5 for dynamic target identification.

**Figure 2:**
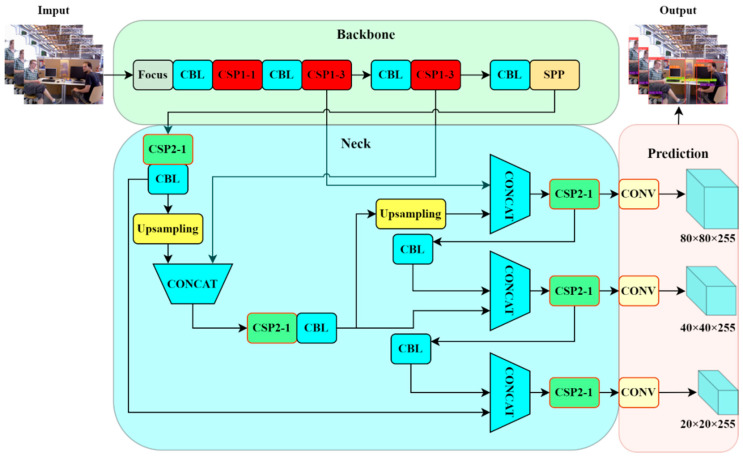
The network architecture diagram of Object Detection.

The YOLOv5 network architecture primarily consists of the Backbone, Neck, and Prediction networks, as shown in Figure 2. The Backbone Network uses CSPDarknet as its framework, combining Focus layers for initial feature extraction and multiple convolutional layers and CSP structures to extract image features. The Neck Network adopts the PANet (Path Aggregation Network) structure to enhance the fusion of features at different scales, thereby improving the model’s detection capability for targets of varying sizes. The Prediction Head Network performs category classification and bounding box regression on multiple scales, utilizing Anchor Boxes to predict the position and size of targets, ensuring effective detection across various scales. The whole structure is designed so that YOLOv5 can not only efficiently extract features from images (Backbone), but also synthesizes these features at different scales for effective target detection (Neck), and ultimately accurately predict the target’s category and location (Prediction).

**Figure 3:**
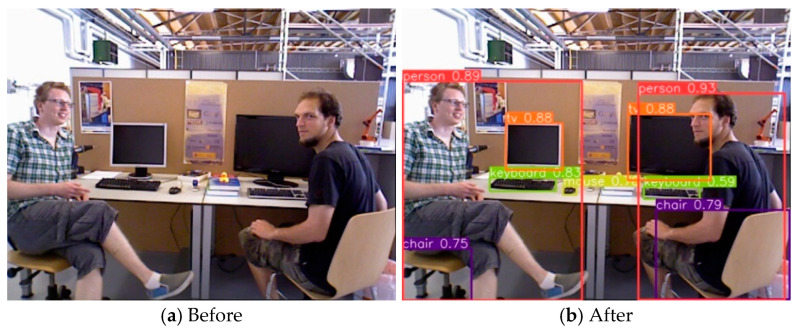
Results of Target Detection in Dynamic Scenes.

Since BMP-SLAM is primarily meant for inside dynamic scenes, we pre-train YOLOv5 with the COCO [31] dataset, which contains the bulk of common interior items. The detection results are achieved, as shown in Figure 3. As seen in Figure 3a, an RGB camera captures a picture sequence that is used as an input to YOLOv5 in this paper. Following Yolov5 processing, the outputs are shown in Figure 3b, where it is clear that distinct objects yield varied-sized anchor frames along with labelling information.

However, after target detection by YOLOv5, although the surrounding environment object categories can be determined, dynamic and static objects cannot be determined. So we define three types of sets for some common objects indoors. High-dynamic objects, such as humans, cats, dogs, and other animals, may move actively. Medium-dynamic objects, such as remote controls, mobile phones, and bags, may move, but their movement is typically driven by external forces rather than autonomous motion. Low dynamic objects, such as monitors, refrigerators, tables, etc., do not move under normal conditions. Dynamic and static objects are identified by YOLOv5 by determining the object categories of the surroundings and then combining these three categories of a priori knowledge for category matching.

**Figure 4:**
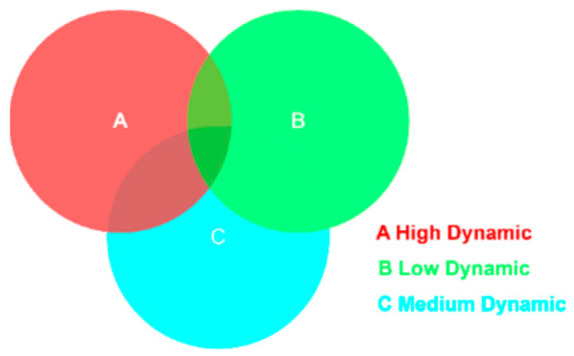
Schematic diagram of dynamic regional judgement.

Despite BMP-SLAM’s capability to identify dynamic regions following target detection and processing of a priori information, a challenge arises due to the nature of YOLOv5’s target detection framework, which encompasses the entirety of objects. This can lead to overlaps in the detection frames of different objects during the detection process. Particularly, removing all keypoints within the detection frame of a highly dynamic object might result in the loss of critical information, adversely affecting the localization accuracy of the BMP-SLAM system. To address this issue, BMP-SLAM includes a preprocessing step that refines dynamic area determination. This stage involves identifying a region as dynamic only if the keypoints in the current frame fall within the bounding box of a high-dynamic item while remaining outside those of low-dynamic objects. Figure 4 depicts a schematic diagram of the dynamic region judging procedure.

**Figure 5:**
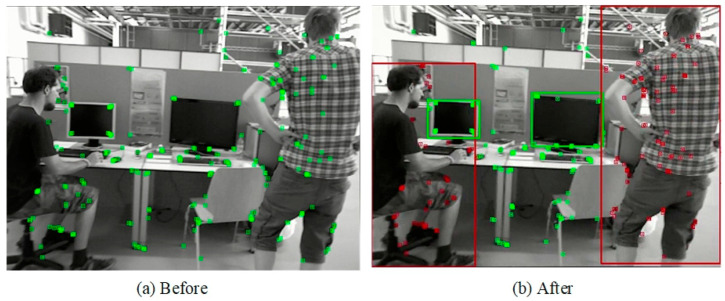
Diagram of Dynamic Area Determination Results, the red is the detection box for high-dynamic object targets, and the green is the detection box for low-dynamic object targets.

Following the described processing steps, dynamic areas within the scene are accurately identified, as demonstrated in Figure 5. Figure 5a displays the original, unprocessed image, while Figure 5b reveals the outcomes of post-processing. In these figures, green dots signify static feature points, and red dots designate dynamic feature points. Additionally, red rectangles encapsulate high-dynamic objects, whereas green rectangles outline low-dynamic objects. Notably, low-dynamic objects, such as computers, initially appear within the detection frames of high-dynamic objects, like people. However, the processed results distinctively segregate dynamic from static regions: feature points on high-dynamic entities are correctly marked as dynamic, whereas those on low-dynamic items, exemplified by the computer, are marked as static. This precise delineation ensures that the dynamic region excludes low-dynamic objects, thereby enhancing the accuracy of dynamic feature point identification.

### 3.3. Bayesian Moving Probability Model

The direct application of YOLOv5 to dynamic object recognition may lead to problems like over-sensitivity to anomalies and under-utilization of historical data. In order to overcome the limitations of YOLOv5, this paper suggests the Bayesian Moving Probability Model, which combines historical data and time-series data to ensure that the dynamic estimation of the key point is not dependent just on a single observation. This improves the VSLAM system’s comprehension of the dynamic environment.

In this paper, we define the dynamic probability of the key point in an image frame F at a given moment *t* as $P\left(m_{t}=d\right)$ and the static probability as $P\left(m_{t}=s\right)$. The initial dynamic and static probabilities of the key points are set to 0.5. After the dynamic target detection thread, there are observation probabilities $P\left(z_{t}=d\right)=1$ and $P\left(z_{t}=s\right)=0$ if the critical point is located in the dynamic region, $P\left(z_{t}=d\right)=0$ and $P\left(z_{t}=s\right)=1$ if it is located outside the dynamic region. Since in complex environments, there may be noise or possibly target detection model detection errors, in order to reduce the impact of this situation, we propose an observation model for the critical point state.

(1) $$\left\{\begin{array}{c} P\left(z_{t}=d|m_{t}=d\right)=\lambda \\ P\left(z_{t}=s|m_{t}=d\right)=1-\lambda \\ \begin{array}{c} P\left(z_{t}=s|m_{t}=s\right)=\omega \\ P\left(z_{t}=d|m_{t}=s\right)=1-\omega \end{array} \end{array}\right.$$

In Equation (1), λ represents the probability of accurately observing a key point as dynamic in a dynamic environment, while 1−λ represents the probability of a key point being misclassified as static in a dynamic environment. ω denotes the probability that a critical point is observed to be static in a static environment, while 1−ω refers to the probability that a critical point is misclassified as dynamic in a static environment. To reduce the impact of such erroneous observations, we set the correct dynamic probability  λ and static probability ω  to 0.9, and the incorrect static probability 1−λ and dynamic probability 1−ω to 0.1.

We consider the motion probability $P_{t-1}\left(X_{i}\right)$ of key point i in the previous frame as the prior probability. This is followed by solving for the observation probability through the dynamic object detection thread and observation model, and then updating the posterior probability (the motion probability of the key point) of each key point according to Bayes’ theorem. Assume that $m_{t}$ denotes the key point state of the current frame and $z_{t}$ denotes the observation of the current frame. Therefore, the motion probability $P_{t}\left(X_{i}\right)=P\left(m_{t}|z_{t}\right)$ of the current frame key point i. The initial dynamic probability $P\left(m_{t}=d\right)=0.5$ and static probability $P\left(m_{t}=s\right)=0.5$ for the current frame key point.

(2) $$P\left(m_{t}=d|z_{t}\right)=\frac{P\left(z_{t}|m_{t}=d\right)P\left(m_{t}=d\right)}{P\left(Z_{t}\right)}$$

(3) $$P\left(m_{t}=s|z_{t}\right)=\frac{P\left(z_{t}|m_{t}=s\right)P\left(m_{t}=s\right)}{P\left(Z_{t}\right)}$$

In Equations (2) and (3), is calculated according to the full probability formula:

(4) $$P\left(z_{t}\right)=P\left(z_{t}|m_{t}=d\right)P\left(m_{t}=d\right)+P\left(z_{t}|m_{t}=s\right)P\left(m_{t}=s\right)$$

The key point is in the dynamic region and the motion probability of the key point is:

(5) $$P\left(m_{t}=d|z_{t}=d\right)=\frac{\lambda \cdot P_{t-1}\left(X_{i}=d\right)}{\lambda \cdot P_{t-1}\left(X_{i}=d\right)+\left(1-\omega \right)\cdot P_{t-1}\left(X_{i}=s\right)}$$

(6) $$P\left(m_{t}=s|z_{t}=d\right)=\frac{\left(1-\omega \right)\cdot P_{t-1}\left(X_{i}=s\right)}{\lambda \cdot P_{t-1}\left(X_{i}=d\right)+\left(1-\omega \right)\cdot P_{t-1}\left(X_{i}=s\right)}$$

The key point is located in the static region and the motion probability of the key point is:

(7) $$P\left(m_{t}=d|z_{t}=d\right)=\frac{\lambda \cdot P_{t-1}\left(X_{i}=d\right)}{\lambda \cdot P_{t-1}\left(X_{i}=d\right)+\left(1-\omega \right)\cdot P_{t-1}\left(X_{i}=s\right)}$$

(8) $$P\left(m_{t}=s|z_{t}=s\right)=\frac{\omega \cdot P_{t-1}\left(X_{i}=s\right)}{\left(1-\lambda \right)\cdot P_{t-1}\left(X_{i}=d\right)+\omega \cdot P_{t-1}\left(X_{i}=s\right)}$$

**Figure 6:**
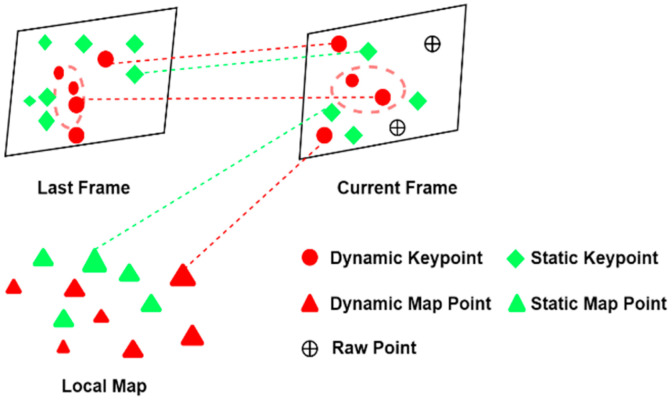
Moving Probability model.

By calculation, if the motion probability of the current key point is greater than a set threshold, it is eliminated so that the ORB-SLAM3 algorithm does not retain them in the later stages of the pose tracking and mapping process. If less than the set threshold, continue with the propagation of dynamic probability, using the posterior probability of the current moment as the prior probability for the next moment. The specific details of propagation are illustrated in the diagram, where the red dashed circles in Figure 6 indicate dynamic areas. The larger the key points in the diagram, the higher their dynamic probability.

### 3.4. Dense Point Cloud Reconstruction

The maps constructed by ORB-SLAM3 mainly rely on spatial geometric features, which are achieved by extracting and tracking ORB feature points in RGB images. However, this feature-point dependent approach faces specific challenges in dynamic environments. In dynamic environments, the movement of moving objects may create ghosting or overlapping effects on the map, thereby limiting the performance of ORB-SLAM3 in dynamic scenes. In addition, since ORB-SLAM3 mainly constructs feature point-based maps, this results in relatively sparse generated maps, which may not be sufficiently effective in complex robot navigation tasks that require high-density map information.

**Figure 7:**
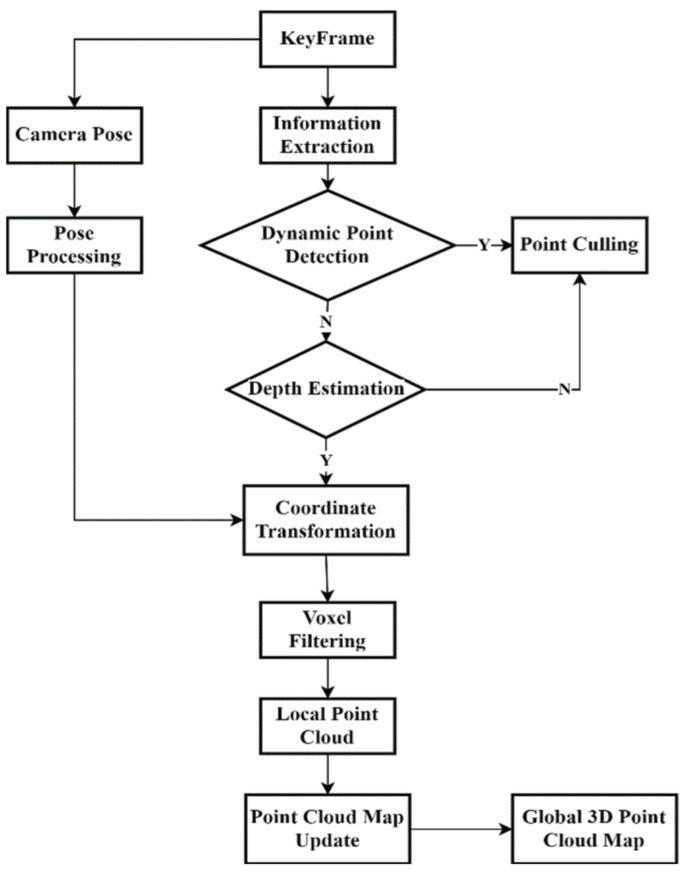
Flowchart of dense point cloud map reconstruction.

To address the above issues, we added a dense point cloud reconstruction thread to ORB-SLAM3, aiming to construct a denser and static point cloud map, as shown in Figure 7. This thread uses keyframes and combines them with the acquired semantic information obtained by the added dynamic target detection thread to exclude dynamic objects and generate a dense static point cloud map. This approach significantly improves the performance and accuracy of SLAM algorithms for constructing maps in dynamic environments.

The dense point cloud building threads we have constructed are effective in building stable and detailed static dense point cloud maps, even in dynamic environments. Firstly, we utilize the depth information and RGB image data of keyframes to extract two-dimensional pixel coordinates $\left(u,v\right)$ and depth value d from the RGB image. Next, the dynamic points are rejected based on the judgment of the dynamic probability of 2D-pixel points.

Then, a coordinate transformation is performed according to the camera pinhole model (see Equations (9) and (10) for mathematical expressions) to generate the corresponding point cloud data. Subsequently, since the camera inevitably has overlapping viewpoints during operation, this will lead to a large amount of redundant information between neighbouring keyframes. We filter this redundant information through voxel network filtering to obtain a new local point cloud map. Finally, we stitch the local point clouds generated from each keyframe to end up with a complete global dense point cloud map (see Equation (11) for the mathematical expression).

(9) $$Z\left[\begin{array}{c} u\\ v\\ 1 \end{array}\right]=K\left(RP_{w}+T\right)=\left[\begin{array}{ccc} f_{x} & 0 & C_{x}\\ 0 & f_{y} & C_{y}\\ 0 & 0 & 1 \end{array}\right]\left[R\left[\begin{array}{c} x_{w}\\ y_{w}\\ z_{w} \end{array}\right]+T\right]$$

(10) $$\left\{\begin{array}{l} x_{w}=\frac{Z\left(u-C_{x}\right)}{f_{x}}\\ y_{w}=\frac{Z\left(v-C_{y}\right)}{f_{y}}\\ z_{w}=Z=\frac{d}{s} \end{array}\right.$$

where Z denotes the proportionality factor between the depth value and the actual spatial distance; K denotes the camera internal reference matrix; R denotes the camera rotation matrix; $P_{w}$ denotes the 3D spatial coordinates corresponding to the 2D pixel points; T denotes the translation matrix; $f_{x}$ and $f_{y}$ denote the camera focal lengths in the horizontal and vertical directions, respectively; and $C_{x}$ and $C_{y}$ denote the translations of the pixel coordinates from the imaging plane, respectively.

(11) $$P_{A}=\sum_{i=1}^{n}\left(P_{i}\cdot R_{i}+T_{i}\right)$$

where $P_{A}$ denotes the constructed global dense point cloud map, $P_{i}$ denotes the local point cloud for each keyframe, and the rotation matrix $R_{i}$ and translation matrix $T_{i}$ denote the camera’s bit position.

## 4. Experiment

### 4.1. Experimental Preparation

The TUM RGB-D dataset [32] was used to assess BMP-SLAM’s performance in dynamic situations. To better validate the accuracy, performance and robustness of the system in the presence of dynamic sceneries, four highly dynamic scene sequences are chosen and tested in the TUM RGB-D dataset in this paper. The four sequences are fr3_walking_xyz, fr3_walking_rpy, fr3_walking_static, and fr3_walking_halfsphere, where fr3 represents the symbol of the sensor. Walking indicates that the environment is a motion scene. static, rpy, xyz and half sphere indicate four different motion states relative to the sensor.

The CPU model of the computer used for the experiment is Intel Core i5-12400F, the graphics card model is NVIDIA GeForce RTX 3060, and the system environment is ubuntu18.04.

### 4.2. Analysis of Results

BMP-SLAM is mostly improved on the foundation of ORB-SLAM3; thus, we use ORB-SLAM3 as a baseline to analyze, compare, and test BMP-SLAM’s accuracy and resilience in dynamic situations. The absolute trajectory error (ATE), which can be used to visually represent algorithm accuracy and trajectory global consistency, is the direct difference between the estimated and true position. Relative attitude error (RPE) focuses on the relative attitude changes between consecutive frames and can more effectively reflect the algorithm’s ability to model and track the dynamic environment. Consequently, the dynamic SLAM performance is assessed in this research using relative pose error (RPE) and absolute trajectory error (ATE) as metrics. Four metrics of absolute trajectory error (ATE) and relative pose error (RPE) were chosen for this study: root mean square error (RMSE), standard deviation (Std), mean (Mean), and median (Median). The results are shown in Table 2 and Table 3.

Table 2 and Table 3 show that the four metrics of absolute trajectory error and relative attitude error for BMP-SLAM are significantly lower than the corresponding values of ORB-SLAM3 in the TUM dynamic scene sequence dataset after eliminating dynamic objects. The average improvement in RMSE, Std, Mean and Median of absolute trajectory errors in these four-tum high dynamic sequence datasets is 93.34%, 92.69%, 93.53% and 93.14% respectively. The average improvement in RMSE, Std, Mean and Median relative to attitude error was 93.48%, 94.57%, 92.74% and 85.45% respectively. In particular, the BMP-SLAM achieves up to 96% RMSE improvement in the freiburg_3_walking_static dataset. This indicates that, as compared to ORB-SLAM3, BMP-SLAM performs noticeably better in dynamic situations, allowing for more accurate and stable tracking of the camera’s trajectory.

**Figure 8:**
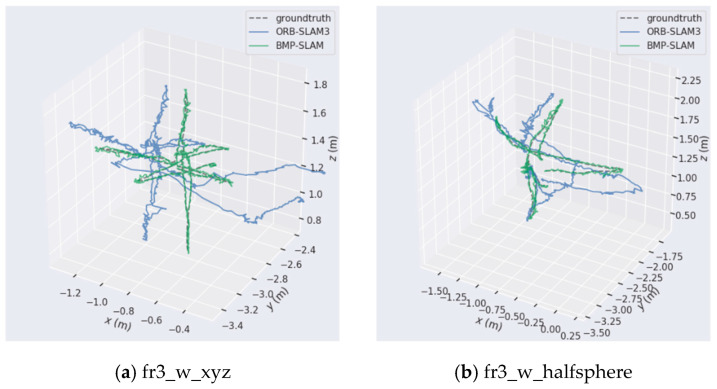
Three-dimensional trajectory comparison map of high dynamic range scenes.

**Figure 9:**
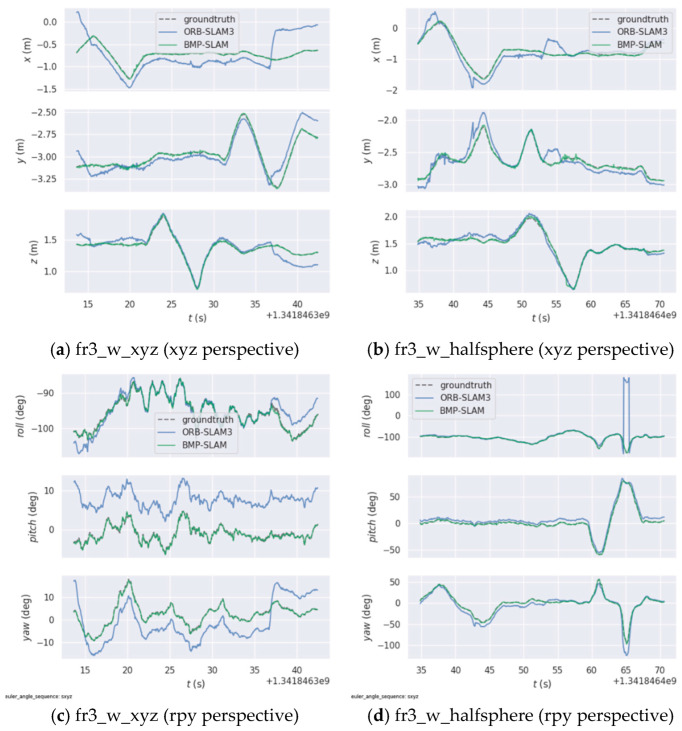
Comparison of dynamic scene trajectories.

We constructed and displayed dynamic scene trajectory comparison maps in xyz and rpy coordinate systems, as well as 3D trajectory comparison maps, to more easily compare the robustness and localization accuracy of BMP-SLAM and ORB-SLAM3 in dynamic environments (As shown in Figure 8 and Figure 9). The three-dimensional trajectory distributions for the fr3_walking_xyz and fr3_walking_halfsphere datasets are shown in Figure 8a and Figure 8b, respectively. Whereas the green solid line represents the trajectory calculated by BMP-SLAM, the blue solid line represents the trajectory estimated by ORB-SLAM3, and the black dashed line represents the genuine trajectory. All trajectories are in metres (m) on the x, y and z axes. The trajectory distributions for the xyz and rpy views under the fr3_walking_xyz dataset are displayed in Figure 9a and Figure 9c, respectively, and for the xyz and rpy views under the fr3_walking_halfsphere dataset are displayed in Figure 9b,d. The real trajectory is represented by the black dashed line in Figure 9, the estimated trajectory by ORB-SLAM3 is represented by the blue solid line, and the estimated trajectory by BMP-S that the estimated trajectories of ORB-SLAM3 deviate significantly from the true trajectories in some regions, which suggests that ORB-SLAM3 may suffer from a lack of accuracy when in a dynamic environment. In contrast, the green solid line indicates the trajectory estimated by BMP-SLAM, which overlaps significantly more with the real trajectory, especially in the region of turns and complex motion patterns.

Figure 9a,b show the positional changes in the three spatial dimensions of x, y and z, respectively. It can be observed that the gap between the green line (the estimated trajectory of BMP-SLAM) and the black dashed line (the true trajectory) is smaller, suggesting that BMP-SLAM is able to track the true position more accurately. Conversely, the blue line of ORB-SLAM3 deviates more at some time points, especially on the Y and Z axes. Figure 9c,d display the changes in three rotation angles over time. The green line (estimated trajectory by BMP-SLAM) closely aligns with the black dashed line (actual trajectory), indicating that BMP-SLAM has higher accuracy in angle estimation. Combining the depictions in Figure 8 and Figure 9, it can be clearly confirmed that in dynamic environments, BMP-SLAM shows superior accuracy and enhanced robustness in the estimation of both spatial position and attitude angle, which is significantly better than the conventional ORB-SLAM3 algorithm.

### 4.3. Ablation Experiment

In this paper, we present the Bayesian Moving Probability Model to address the inadequacies of the one-stage target detection algorithm’s yolov5 model and increase the accuracy and resilience of the ORB-SLAM3 system in dynamic situations. To verify the validity of this model, we design this ablation experiment to quantify its effect on the overall system. In this part of the experiment, we use ORB-SLAM3 as a benchmark to compare the absolute trajectory error and relative attitude error before and after adding the Bayesian Moving Probability Model. The test results are shown in Table 4 and Table 5.

According to the results in Table 4 and Table 5, it is shown that ORB-SLAM3, after adding the dynamic table detection thread constructed in this paper, eliminates the feature points located in the dynamic region and thus does not participate in the system’s later attitude tracking and mapping process as a way to improve the system’s accuracy and robustness in dynamic environments. However, the enhancement effect is limited because dynamic target detection threads are likely to misdetect or omit detection even in the face of complex dynamic environments. When the Bayesian Moving Probability Model was fused in the tracking thread, the accuracy in the highly dynamic sequence fr3_walking_xyz dataset was improved by 50% compared to before the fusion, and the overall accuracy was improved by 95.64%.

**Figure 10:**
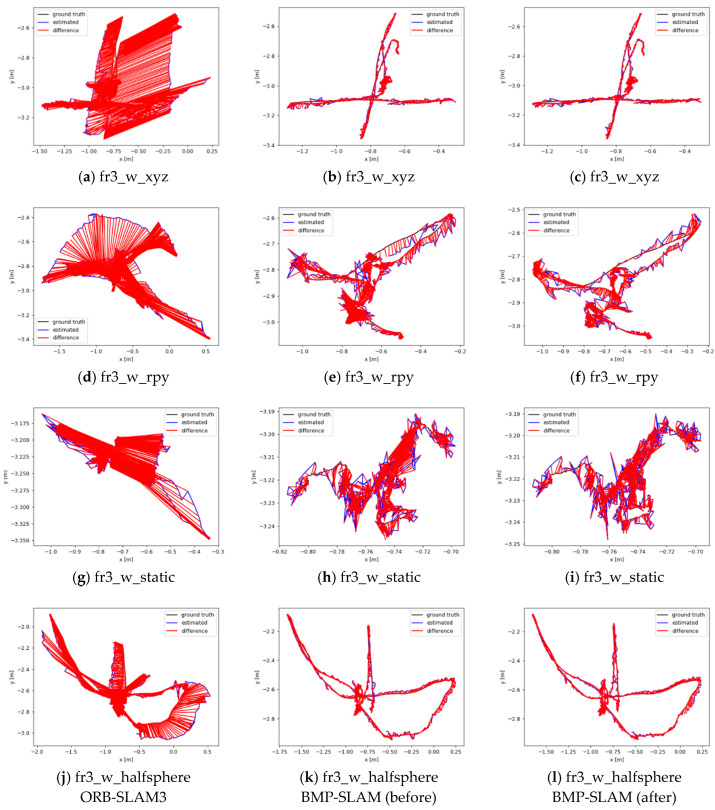
Comparison of trajectory errors before and after the addition of the Bayesian Moving Probability Model, (**a**,**d**,**g**,**j**) represent the original ORB-SLAM3 system; (**b**,**e**,**h**,**k**) represent before the addition of the Bayesian Moving Probability Model; (**c**,**f**,**i**,**l**) represent after the addition of the Bayesian Moving Probability Model.

**Figure 11:**
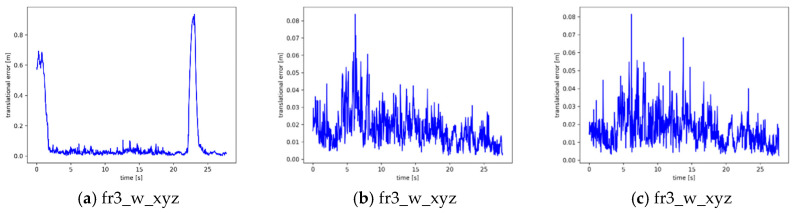

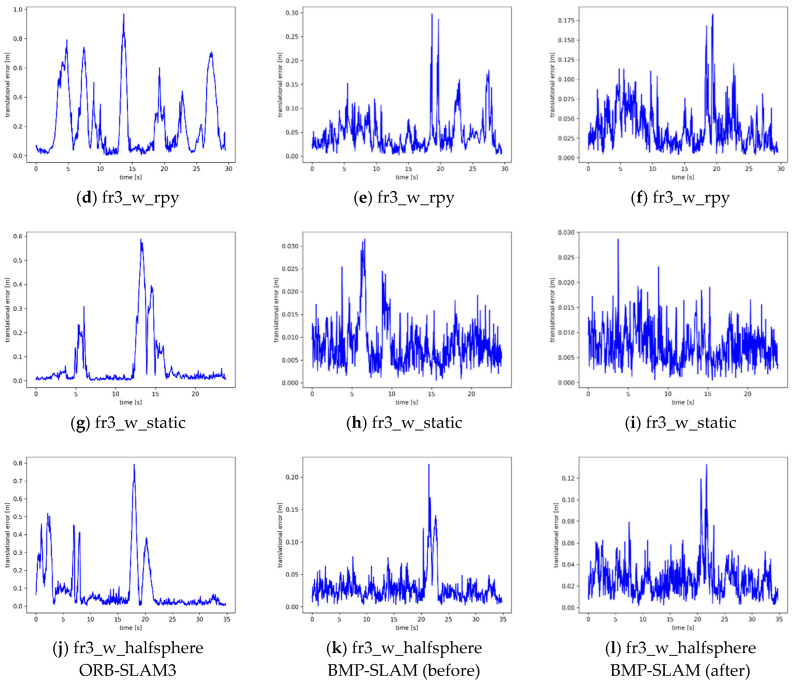
Comparison of time series errors before and after the addition of the Bayesian Moving Probability Model, (**a**,**d**,**g**,**j**) represent the original ORB-SLAM3 system; (**b**,**e**,**h**,**k**) represent before the addition of the Bayesian Moving Probability Model; (**c**,**f**,**i**,**l**) represent after the addition of the Bayesian Moving Probability Model.

In order to observe the results more intuitively, we also plotted trajectory error comparisons and time series error comparisons based on ATE and RPE, as shown in Figure 10 and Figure 11. where black indicates the actual trajectory and blue indicates the estimated trajectory. The red colour indicates the difference between the estimated trajectory and the actual trajectory. The unit of the horizontal and vertical axes in Figure 10 is m. The unit of the horizontal axis in Figure 11 is s, and the unit of the vertical axis is m. The unit of the horizontal axis in Figure 11 is s, and the unit of the vertical axis is m.

From Figure 10a,d,g,j, it can be seen that ORB-SLAM3 has a large error between the estimated trajectory and the actual trajectory, and the trajectory offset is also serious under the four highly dynamic sequences. After going through the dynamic target detection thread, the reduction of the red region can be visualised very well according to Figure 10b,e,h,k, which indicates the reduction of the error between the estimated trajectory and the actual trajectory. Figure 10c,f,i,l represents the performance of our method after fully integrating the Bayesian Moving Probability Model. As can be seen in Figure 10, the error between the estimated trajectory and the actual trajectory is further reduced, and the trajectory offset phenomenon is also significantly improved. This indicates that the Bayesian Moving Probability Model significantly improves the accuracy and robustness of the system in dynamic environments.

Figure 11a,d,g,j shows the RPE of the conventional ORB-SLAM3 system on different dynamic sequence datasets. It can be seen that the distribution interval of the RPE is very wide in some cases, especially in Figure 11a,d, with peaks as high as about 0.9 m, which suggests that there is a very serious drift problem of the ORB-SLAM3 system in a dynamic environment. Figure 11b,e,h,k shows the performance of the system before the integration of the Bayesian Moving Probability Model. Compared to ORB-SLAM3, the peaks of RPE are reduced in most cases, especially in Figure 11e, which suggests that the system also achieves some performance gains after adding the dynamic target detection thread.

However, the distribution of the RPE is still not uniform, especially at some moments in Figure 11b, with an error of 0.08 m, showing some extreme errors. Figure 11c,f,i,l shows the system after integrating the Bayesian Moving Probability Model. The peaks in RPE were generally low in all sequences, indicating that the addition of the Bayesian motion probability propagation model significantly improved the accuracy of the system. Especially in Figure 11f,l, the maximum peak of RPE was significantly reduced. It is worth noting that by comparing Figure 11e,f, it can be found that the RPE error distribution interval before integrating the Bayesian Moving Probability Model is mainly in the interval from 0.01 m to 0.15, with peaks up to 0.3 m. After integrating the Bayesian Moving Probability Model, the distribution interval is mainly in the interval of 0.01 m to 0.075, and the peak value is only 0.15 m at the highest, which indicates that the performance of the system in the dynamic scene has been significantly improved after integrating the Bayesian Moving Probability Model.

### 4.4. Dynamic SLAM Comparison Experiment

To further validate the performance of the proposed method in dynamic environments, this section conducts comparative evaluations using RGB-D data from the TUM dynamic scene dataset, benchmarking against state-of-the-art dynamic SLAM algorithms including DS-SLAM, DynaSLAM, and Blitz-SLAM. The experiments employ Absolute Trajectory Error (ATE) and Relative Pose Error (RPE) as evaluation metrics to comprehensively analyze the localization accuracy and robustness of each algorithm. Quantitative evaluation results across different sequences are presented in Table 6 and Table 7.

As demonstrated in Table 6 and Table 7, the proposed BMP-SLAM algorithm achieves significantly superior performance compared to DS-SLAM in high-dynamic scenarios, with an average reduction of 39.39% in ATE. The most significant improvement was observed in the fr3_walking_rpy sequence, where our BMP-SLAM algorithm reduced the RMSE from 0.4442 m (DS-SLAM) to 0.0339 m, representing a remarkable 92.37% enhancement in localization accuracy, as shown in Table 6. The proposed BMP-SLAM algorithm demonstrates superior performance in RPE across all tested scenarios except for the fr3_walking_rpy sequence, where DS-SLAM achieves marginally better results. The most significant RMSE improvement was achieved in the fr3_walking_rpy sequence, where our BMP-SLAM algorithm reduced the error from 0.1503 m (DS-SLAM) to 0.0483 m, representing a 67.86% reduction, as shown in Table 7. Since DS-SLAM is based on the lightweight semantic segmentation network SegNet, it determines the state of feature points based on the feature information of the adjacent two frames. Therefore, its performance for low-dynamic scene datasets is superior to that of the BMP-SLAM algorithm proposed in this paper. The proposed BMP-SLAM algorithm demonstrates consistent superiority over DynaSLAM in high-dynamic scenarios, achieving an average 5.09% reduction in ATE across all tested sequences. In the sequence of low dynamic scene datasets, the performance of DynaSLAM is slightly better than that of the BMP-SLAM algorithm proposed in this paper. This is mainly attributed to the fact that DynaSLAM uses Mask R-CNN for instance segmentation, which can obtain precise pixel-level semantic segmentation and instance labels, thereby identifying and eliminating dynamic feature points more accurately. Compared with Blitz-SLAM, in the high dynamic scene dataset, except for the performance in the fr3_walking_xyz scene, which is slightly better than that of the BMP-SLAM in this paper, the performance of the BMP-SLAM in this paper is better than Blitz-SLAM in other datasets. In summary, the proposed BMP-SLAM algorithm demonstrates superior performance in the majority of high-dynamic scenarios compared to other methods, while maintaining comparable performance in low-dynamic environments.

**Figure 12:**
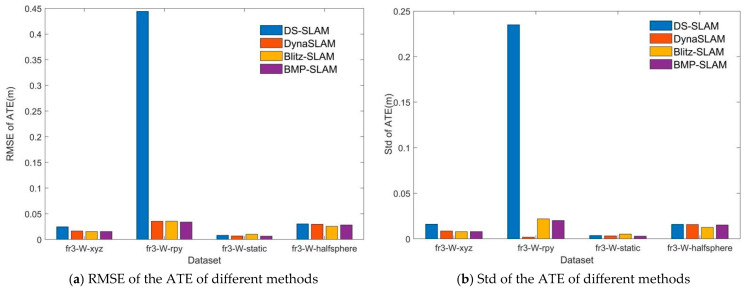
RMSE and Std of ATE of different methods under different datasets.

Based on the above analysis, we obtained the RMSE and Std of the ATE of different methods, as shown in Figure 12.

For the fr3_W_xyz scene, it can be clearly seen from Figure 12a,b that the RMSE and Std of the ATE obtained by the BMP-SLAM algorithm proposed in this paper are similar to those of DynaSLAM and Blitz-SLAM, and both are slightly lower than the DS-SLAM method. As for the fr3_W_rpy scene, it can be seen from Figure 12a that the RMSE of the ATE obtained by the method in this paper is similar to DynaSLAM and Blitz-SLAM, and both are much lower than the DS-SLAM method. It can be seen from Figure 12b that the Std of the ATE of the proposed method in this paper is similar to Blitz-SLAM, but greater than the DynaSLAM method. For the fr3_W_static scene, the RMSE and Std of the ATE of the method proposed in this paper are slightly lower than those of the other three methods. In the case of the fr3_W_halfsphere scene, all four methods demonstrate comparable performance in terms of both RMSE and Std of ATE.

### 4.5. Dense Point Cloud Reconstruction Experiment

**Figure 13:**
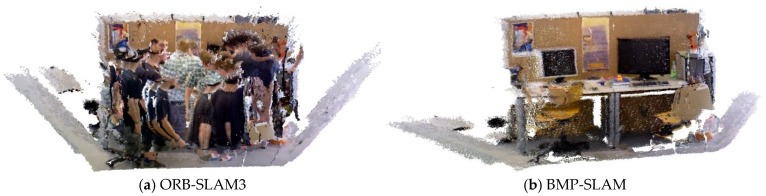
Dense point cloud reconstruction.

To evaluate the performance of the proposed BMP-SLAM to generate dense point cloud maps in dynamic situations, we added dense map construction threads to ORB-SLAM3 as a control group for comparative tests. In this experiment, we used the highly dynamic scene data sequence fr3_walking_xyz from the TUM RGB-D dataset, which was tested, and the test results are shown in Figure 13. Figure 13a shows the dense point cloud map generated by the original ORB-SLAM3 algorithm in a dynamic environment. It can be clearly observed that the presence of dynamic objects in the environment leads to severe ghosting in the map, which is mainly caused by the difficulty of the SLAM system to accurately track all the feature points when the dynamic objects are moving rapidly. Figure 13b shows the dense point cloud map constructed by BMP-SLAM. From the comparative analysis of Figure 13a,b, it can be intuitively observed that the interference caused by dynamic targets is effectively cut down, and the point cloud presents higher stability and continuity in most areas. This result validates the improved accuracy of our constructed dynamic target detection thread for map construction in dynamic environments.

However, in Figure 13b, the point cloud in some regions appears to be empty, which is due to the excessive size of the target detection frame in the dynamic target detection thread that causes some static feature points to be wrongly rejected, which in turn causes the loss of some feature information. Despite this problem, objects in the environment can still be clearly recognised, as shown in Figure 13b. In turn, it can satisfy complex robot navigation tasks with high-density information requirements.

### 4.6. Real-Time Performance Analysis

In SLAM research, the real-time performance of algorithms serves as one of the critical metrics for evaluating their effectiveness. To accurately assess and compare the real-time capability of our proposed algorithm, this section presents a comparative analysis of the average per-frame tracking time consumption among ORB-SLAM3, DS-SLAM, DynaSLAM, Blitz-SLAM, and our method. As clearly demonstrated in Table 8, DynaSLAM exhibits the longest average processing time, exceeding 300 ms per frame. This indicates significant limitations in real-time image processing, primarily due to its high computational complexity, which may demand substantial computational resources. The main reason is that DynaSLAM uses the Mask R-CNN instance segmentation algorithm to detect and filter dynamic targets. The algorithm complexity is relatively high, so the accuracy is high, but the consumption time is relatively long. In contrast, DS-SLAM employs the lightweight semantic segmentation network SegNet, which reduces computational time compared to DynaSLAM. However, its average processing time still exceeds 140 ms, resulting in suboptimal real-time performance. This may limit its applicability in scenarios demanding strict real-time constraints. Blitz-SLAM utilizes BlitzNet to efficiently obtain bounding boxes and masks of dynamic objects, enabling rapid separation between static backgrounds and dynamic regions in the image. This approach significantly improves real-time performance compared to previous methods, with an average time consumption of 81ms, as shown in Table 8. However, these methods all rely on semantic segmentation approaches, essentially trading computational time for improved accuracy. In contrast, our method employs object detection, which maintains superior real-time performance with an average processing time of 60 ms—significantly lower than the three comparative methods—while still achieving high precision.

## 5. Conclusions

In this paper, we propose a target detection-based VSLAM algorithm (BMP-SLAM) for indoor dynamic scenes, aiming to reduce the impact of dynamic objects on the SLAM system. The proposed BMP-SLAM in this study builds upon ORB-SLAM3 by incorporating an improved YOLOv5 object detection algorithm, which establishes a dedicated dynamic object detection thread to capture environmental semantic information and identify dynamic regions. In addition, the Bayesian Moving Probability Model is integrated into the tracking thread for data correlation, which in turn achieves effective culling of dynamic feature points and significantly improves the accuracy and robustness of the system in dynamic environments. BMP-SLAM also combines keyframe information and semantic information provided by the dynamic target detection thread to perform dynamic object rejection, enabling dense point cloud map construction for indoor 3D scenes. In order to evaluate the BMP_SLAM performance, four highly dynamic scene sequences in the TUM RGB-D dataset were selected for extensive experiments. Experimental findings demonstrate that BMP-SLAM significantly outperforms ORB-SLAM3 in dynamic environments. Moreover, the ATE and RPE of the proposed BMP_SLAM are significantly lower than those of DS-SLAM and slightly lower than those of DynaSLAM and Blitz-SLAM methods, achieving more stable and precise camera trajectory tracking. Even when compared with other excellent dynamic SLAM methods, it possesses smaller trajectory error and more accurate localisation accuracy, which further validates the robustness of BMP-SLAM. In addition, the dense point cloud map constructed by BMP-SLAM can also effectively meet the requirements of indoor complex robot navigation tasks.

Although BMP-SLAM performs well in dynamic indoor environments, there are some limitations that we will try to address in future work. Since the algorithm relies on YOLOv5 for dynamic object detection, it shows a strong dependency on the training dataset. So it leads to the fact that the removal effectiveness of the algorithm may be affected when there are dynamic objects in the environment that are not defined in the training set. Therefore, the ability to handle unknown dynamic objects needs to be enhanced by incorporating more advanced target detection techniques or adopting unsupervised learning methods to improve the adaptability and robustness of the algorithms in diverse environments.

## Figures and Tables

**Table 1:** Comparison of the advantages and disadvantages of SLAM algorithms in dynamic environments.

	Based on Geometric Methods	Based on Deep Learning Methods	
		Semantic Segmentation	Object Detection
Strengths	High universality, efficient, capable of autonomous adaptation in unknown environments, and not dependent on prior knowledge.	Accurately identify moving objects.	High speed, good real-time performance.
Weaknesses	In complex dynamic scenes, it is not possible to effectively distinguish between moving and stationary objects.	Slow speed, unable to run in real time.	Overly dependent on the target detection network

**Table 2:** Comparison of TUM dynamic sequence ATEtests (Unit: m).

Sequence	ORB-SLAM3				BMP-SLAM			
	RMSE	Std	Mean	Median	RMSE	Std	Mean	Median
W_xyz	0.3547	0.1917	0.2984	0.2121	0.0155	0.0079	0.0133	0.0118
W_rpy	0.5363	0.2229	0.4877	0.4426	0.0339	0.0201	0.0272	0.0211
W_static	0.1753	0.0827	0.1545	0.1711	0.0064	0.0029	0.0057	0.0053
W_halfsphere	0.2299	0.1204	0.1959	0.1435	0.0282	0.0152	0.0238	0.0202

**Table 3:** Comparison of TUM dynamic sequence RPE tests (Unit: m).

Sequence	ORB-SLAM3				BMP-SLAM			
	RMSE	Std	Mean	Median	RMSE	Std	Mean	Median
W_xyz	0.5069	0.3819	0.3334	0.0480	0.0219	0.0103	0.0194	0.0178
W_rpy	0.7820	0.4224	0.6581	0.6576	0.0483	0.0263	0.0405	0.0340
W_static	0.2489	0.1506	0.1982	0.1967	0.0098	0.0044	0.0087	0.0081
W_halfsphere	0.3401	0.2067	0.2701	0.2819	0.0395	0.0196	0.0342	0.0311

**Table 4:** Comparison of ATE before and after adding Bayesian Moving Probability Model (Unit: m).

Sequence	ORB-SLAM3		Before		After	
	RMSE	Std	RMSE	Std	RMSE	Std
W_xyz	0.3547	0.1917	0.0164	0.0082	0.0155	0.0079
W_rpy	0.5363	0.2229	0.0549	0.0327	0.0339	0.0201
W_static	0.1753	0.0827	0.0087	0.0045	0.0064	0.0029
W_halfsphere	0.2299	0.1204	0.0313	0.0176	0.0282	0.0152

**Table 5:** Comparison of RPE before and after adding Bayesian Moving Probability Model (Unit: m).

Sequence	ORB-SLAM3		Before		After	
	RMSE	Std	RMSE	Std	RMSE	Std
W_xyz	0.5069	0.3819	0.0236	0.0112	0.0219	0.0103
W_rpy	0.7820	0.4224	0.0780	0.0467	0.0483	0.0263
W_static	0.2489	0.1506	0.0129	0.0063	0.0098	0.0044
W_halfsphere	0.3401	0.2067	0.0447	0.0231	0.0395	0.0196

**Table 6:** Comparison of ATE Tests of Different Algorithms (Unit: m).

Dataset	DS-SLAM		DynaSLAM		Blitz-SLAM		Ours (BMP-SLAM)	
	RMSE	Std	RMSE	Std	RMSE	Std	RMSE	Std
fr3_W_xyz	0.0247	0.0161	0.0164	0.0086	0.0153	0.0078	0.0155	0.0079
fr3_W_rpy	0.4442	0.2350	0.0354	0.0019	0.0356	0.0220	0.0339	0.0201
fr3_W_static	0.0081	0.0036	0.0068	0.0032	0.0102	0.0052	0.0064	0.0029
fr3_W_halfsphere	0.0303	0.0159	0.0296	0.0157	0.0256	0.0126	0.0282	0.0152
fr3_S_xyz	-	-	0.0085	0.0045	0.0148	0.0069	0.0207	0.0090
fr3_S_static	0.0065	0.0033	0.0108	0.0056	-	-	0.0110	0.0053

**Table 7:** Comparison of RPE Tests of Different Algorithms (Unit: m).

Dataset	DS-SLAM		DynaSLAM		Blitz-SLAM		Ours (BMP-SLAM)	
	RMSE	Std	RMSE	Std	RMSE	Std	RMSE	Std
fr3_W_xyz	0.0333	0.0229	0.0217	0.0119	0.0197	0.0096	0.0219	0.0103
fr3_W_rpy	0.1503	0.1168	0.0448	0.0262	0.0473	0.0283	0.0483	0.0263
fr3_W_static	0.0102	0.0038	0.0089	0.0044	0.0129	0.0069	0.0098	0.0044
fr3_W_halfsphere	0.0297	0.0152	0.0284	0.0149	0.0253	0.0136	0.0395	0.0196
fr3_S_xyz	-	-	0.0142	0.0073	0.0144	0.0071	0.0207	0.0090
fr3_S_static	0.0078	0.0038	0.0126	0.0067	-	-	0.0110	0.0053

**Table 8:** Time consumption (Unit: s).

Different Methods	GPU	Model	Time-Consuming (ms)
ORB-SLAM3	-	-	15.125
DS-SLAM	P4000	SegNet	>140
DynaSLAM	NVIDIA Tesla M40 GPU	Mask R-CNN	>300
Blitz-SLAM	-	BlitzNet	81
Ours	NVIDIA GeForce RTX 3060	Yolov5	60

## Data Availability

The data used to support the findings of this study are available from the corresponding author upon request.
